# Age Shall Not Weary Us: Deleterious Effects of Self-Regulation Depletion Are Specific to Younger Adults

**DOI:** 10.1371/journal.pone.0026351

**Published:** 2011-10-24

**Authors:** Theresa Dahm, Hamid Taher Neshat-Doost, Ann-Marie Golden, Elizabeth Horn, Martin Hagger, Tim Dalgleish

**Affiliations:** 1 Medical Research Council Cognition and Brain Sciences Unit, Cambridge, United Kingdom; 2 University of Isfahan, Isfahan, Iran; 3 University of Nottingham, Nottingham, United Kingdom; University of Leicester, United Kingdom

## Abstract

Self-regulation depletion (SRD), or ego-depletion, refers to decrements in self-regulation performance immediately following a different self-regulation-demanding activity. There are now over a hundred studies reporting SRD across a broad range of tasks and conditions. However, most studies have used young student samples. Because prefrontal brain regions thought to subserve self-regulation do not fully mature until 25 years of age, it is possible that SRD effects are confined to younger populations and are attenuated or disappear in older samples. We investigated this using the Stroop color task as an SRD induction and an autobiographical memory task as the outcome measure. We found that younger participants (<25 years) were susceptible to depletion effects, but found no support for such effects in an older group (40–65 years). This suggests that the widely-reported phenomenon of SRD has important developmental boundary conditions casting doubt on claims that it represents a general feature of human cognition.

## Introduction

Self-control - defined as success in effortfully overriding or altering one's thoughts, emotions, motivations or behaviours [1] – was considered by Aristotle as one of the most difficult things known to humanity: “I count him braver who overcomes his desires than him who overcomes his enemies; for the hardest victory is victory over self.” Difficult as it may be, self-control is quintessential to the successful navigation of daily temptations, as well as the achievement of long-term goals. Self-control improves with practice [2], and also with age [3]. Unsurprisingly, this latter finding corresponds to the maturation of the frontal lobes, which are associated with inhibitory control and continue to develop into early adulthood [4].

The strength or limited resource model of self-control compares the capacity for self-control to the strength of a muscle or of an energy resource, based on the finding that self-regulatory abilities vary within individuals across time [e.g. 5]. An important implication of this muscle analogy is that self-control can become tired or depleted after use. Impairments in self-control would thus be most likely to occur following prior attempts at regulating behaviour. There is now a wealth of data in support of this ‘ego-depletion’ prediction and a recent meta-analysis has found a medium-to-large effect size of depletion on self-control tasks, preceded by other self-control tasks, across more than eighty studies [6].

One striking feature of nearly all such previous studies on ego-depletion is that the samples tend to be selected from university student populations rather than the general population. In fact, of those studies selected for the recent meta-analysis [6], only eleven recruited participants from the general population, compared with 187 samples drawn from student populations. From those that reported participant age, it is clear that the student samples were, unsurprisingly, significantly younger (k = 39; M age = 21.37, SD = 2.80) than those from the general population (k = 10; M age = 33.94, SD = 7.65) (t = 5.10, p<.01). This reliance on undergraduate samples is undoubtedly a feature of much research in psychology [7]. However, in the domain of self-control and mental regulation it raises a potential problem given that neurological maturity for the frontal brain regions that underpin these faculties occurs later [4] than the mean age of the subjects used in the majority of ego-depletion research. This raises a pressing question: Do ego-depletion effects emerge empirically because of the young participant age of the majority of studies? As a preliminary investigation of this question we conducted a moderator analysis on the Hagger et al. meta-analytic data. This revealed a significantly higher ego-depletion effect size for the younger (student) samples (d = 0.67, CI95 [0.623, 0.72], Q(186) = 244.39, p<.01) relative to the older general population samples (d = 0.32, CI95 [0.18, 0.46], Q(10) = 54.68, p<.001).

While this analysis can only be preliminary due to the relatively small and variable general population samples in this set of studies, it nonetheless supports our speculation that ego-depletion may be a phenomenon mainly restricted to youth. To examine this prediction experimentally we therefore compared depletion effects in two age groups: a younger sample of 18–25 years, and an older group of 40–65 years. We chose not to include the age range of 26–39 to eliminate the effects of any individual differences in brain maturation. We used the Color Stroop paradigm [8] to deplete self-regulatory resources a manipulation that has been used in previous research on ego-depletion [9], [10]. To evoke ego-depletion, we used the incongruent version of this task (Color Stroop condition) in which participants name the color of the ink that a color word is written in; for example if the word RED is written in blue ink, the correct response is ‘blue’. This requires inhibiting the automatic inclination to read the written word, and this is especially demanding of self-regulatory capacity [9]. In line with previous research [9], [10], we also had two control conditions: one in which participants completed no other task before the outcome measure (No Stroop condition) and one in which participants simply read aloud the same words as in the Color Stroop but this time the ink color of all words was black (Control Stroop condition). Following these manipulations, participants completed an Autobiographical Memory Task (AMT) as the outcome measure. The AMT task we used tests the extent to which participants can produce specific personal memories (i.e., of a particular event happening on a single day in the past) to a series of cue words that bring to mind periods of time longer than a day. For example, an appropriate response to the cue ‘HOLIDAY’ would be, “I remember the day that we went to Disneyland last summer”, rather than “We had a great holiday last year in Florida”. Self-regulation is required to resist the cue word's tendency to prime non-specific memories in order to generate a suitably specific memory for an event on an identified day. Participants with relatively impoverished self-control tend to make errors on the AMT by responding with inappropriately general (rather than specific) memories [11], [12] as a function of failing to effectively inhibit these generic event-descriptions during autobiographical memory search [13]. Consequently, number of specific memories produced to a set of cue words on the AMT is a demonstrably sensitive [10] measure of ego-depletion effects and there is considerable previous research that has demonstrated that performance on such processing tasks are susceptible to the depleting effects of prior self-control exertion [14].

We predicted an interaction of depletion condition (Color Stroop, Control Stroop, No Stroop) and age group on the number of specific memories successfully retrieved on the AMT. We expected younger participants allocated to the depletion condition (Color Stroop) would retrieve significantly fewer specific memories relative to younger participants in the control conditions (No Stroop, Control Stroop), but no significant effects for depletion condition among older participants.

## Methods

The study and consent procedure were approved by the National Research Ethics Service Committee East of England - Cambridge South. Prior to the study prospective participants were provided with detailed written and oral information about the study. Participants who chose to participate signed a written informed consent form.

### Participants

Eighty-seven community volunteers with self-reported normal color vision and reading ability, and English as a first language, were recruited from the Cognition and Brain Sciences Unit panel. . Participants were recruited from two age groups: 18–25 years (Younger group) and 40–65 years (Older group). Within each age group, participants were randomly allocated to the three experimental conditions: Color Stroop; Control Stroop; and No Stroop.

### Measures

#### The Stroop task [8]

The two Stroop tasks were as described in the Introduction. In the Color Stroop condition the ink colors (RED, GREEN, BLUE and YELLOW) were always incongruent to the semantic meaning of the same four color words used as stimuli. In the Control Stroop condition participants simply had to read aloud the same four color words which were all now printed in black ink. The Stroop tasks were presented on 42×59 cm cards with 120 words (trials) per card. The cards in each condition were identical to each other, except for ink color. Each condition contained equivalent numbers of color words (30 of each color). In the Color Stroop condition, each color word was written 10 times in each incongruent ink color. Participants performed their respective task for 6½ minutes in line with previous studies using the Stroop task to examine self-regulation depletion [6], [10]. The number of trials completed within this time, and the error rates, for each participant were recorded.

#### Autobiographical Memory Task (AMT)

The AMT used was identical to that used by Neshat-Doost et al. [10]. Participants were presented with 15 cue words each associated with a period of time longer than a single day and varying in emotional valence (e.g., *holiday*, *bereavement*, *adolescence*). Participants were given 30 seconds in each case to retrieve a specific autobiographical memory. They were told that the memory should be of something that happened *at a particular time on a particular day*. Examples of acceptable and unacceptable responses were given. Cue words were presented in a separate random order for each participant. Three practice cues were given (*absence, gigantic*, *grass*).

Generated memories were tape-recorded for coding by AG [12]. Memories were coded as specific (events that lasted for a *day or less*), or non-specific (events that lasted for longer periods of time or events that occurred repeatedly over a period of time). Failure to adhere to the time limit, or talking about things that were not memories was classed as *‘no memories’*. Analyses focus on the total number of specific memories generated to the set of words, in line with previous work on mental regulation and the AMT [10], [11], [15]. Inter-rater agreement for the coding of specific memories between AG and HD (*n* = 150 memories) indicated excellent reliability, κ = .91.

### Procedure

Participants were tested individually in a quiet testing environment. Participants in the two Stroop conditions first completed their respective Stroop tasks while the No Stroop participants did nothing. The Stroop participants rated how difficult their Stroop task had been, and all participants completed ratings of happy and sad mood and tiredness, on scales ranging from 0 (*not at all*) to 100 (*extremely*) [9]. Participants then completed the AMT and were then assessed on measures of depression symptoms (Beck Depression Inventory-II [BDI-II]; [16]) and trait anxiety (Spielberger State-Trait Anxiety Inventory, trait scale [STAI]; [17]), both known to influence memory specificity.

## Results

### Participant characteristics

Participant characteristics are presented in Table 1. The younger and older groups did not differ on symptoms of anxiety or depression, *t*s<1.8, *P*s>.08, gender ratio, or educational level, χ^2^s<0.91, *P*s>.60. Similarly, within each age group the participants in the three conditions were comparable on these variables, as well as on age, *P*s>.15.

**Table 1:** Participant Characteristics and Stroop Task Data Across the Three Experimental Conditions and two Age Groups.

	Color Stroop				Control Stroop				No Stroop			
	Younger		Older		Younger		Older		Younger		Older	
	M	*SD*	M	*SD*	M	*SD*	M	*SD*	M	*SD*	M	*SD*
*n*	14		15		17		15		14		12	
Sex (M∶F)	8∶6		5∶10		5∶12		5∶10		4∶10		5∶7	
Education	5∶2∶6		3∶1∶8		5∶2∶10		5∶2∶7		2∶4∶8		4∶1∶5	
BDI-II	7.14	*6.40*	3.93	*3.69*	5.65	*5.10*	4.87	*4.29*	5.57	*3.94*	4.17	*3.13*
STAI-Trait	37.29	*10.10*	31.60	*9.63*	35.41	*9.02*	28.80	*7.66*	34.50	*5.13*	28.42	*5.18*
No. of Stroop Pages	2.90	*0.38*	2.95	*0.39*	7.22	*0.72*	7.05	*0.83*	-	*-*	-	*-*
Stroop Errors	6.36	*4.25*	7.33	*6.25*	1.12	*1.69*	2.47	*2.39*	-	*-*	-	*-*
Stroop Difficulty	58.21	*21.80*	42.07	*21.51*	21.53	*24.44*	9.73	*15.10*	-	*-*	-	*-*
Tired	47.00	*21.17*	23.00	*18.13*	43.18	*27.35*	21.20	*26.51*	46.43	*13.14*	17.27	*19.02*
Happy	56.64	*14.39*	66.00	*16.71*	57.06	*10.16*	57.73	*24.17*	60.71	*15.92*	71.36	*15.18*
Sad	15.21	*11.35*	13.67	*15.41*	16.76	*16.00*	8.80	*11.31*	21.43	*21.16*	10.91	*16.40*

*Note*. M = Male; F = Female; Education = High School∶College Diploma∶University; BDI-II = Beck Depression Inventory-II; STAI = Spielberger State Trait Anxiety Inventory.

### Stroop task performance

Table 1 also records the relevant data from the Stroop tasks. These data verify that the two age groups did not differ on overall executive task performance prior to any effects of depletion. Accordingly, there were no significant age group differences for numbers of Stroop errors or numbers of Stroop trials completed, nor were there any for mood, *t*s<1.38, *P*s>.17. There were also no significant age group by Stroop condition interactions for these Stroop task variables, *F*s<1. Unsurprisingly, within each age group, the Color Stroop group completed fewer trials on the task, made more errors, and reported the task to be more difficult than the Control Stroop group, *t*s>2.80, *P*s<.01, but there were no differences in terms of tiredness, or sad/happy mood, *t*s<1.09, *P*s>.29. Consistent with our study hypothesis, overall the older participants reported finding the Stroop tasks less tiring than did the younger participants, t(59) = 3.80, P<.01, Cohen's d = 0.99, and there was a trend for the Older group to report the tasks as less difficult, *t*(59) = 1.76, *P* = .08, Cohen's d = 0.46.

### Autobiographical Memory Task Performance

**Figure 1:**
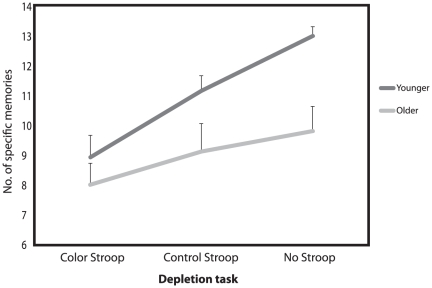
Autobiographical Memory Test performance across conditions. Mean (+1 *SE*) total numbers of specific memories across the Color Stroop, Control Stroop and No Stroop conditions on the Autobiographical Memory Test for the Younger and Older age groups.

Figure 1 shows the total numbers of specific memories retrieved across the three depletion conditions (Color Stroop, Control Stroop, No Stroop) for the two age groups (younger, older). A mixed model ANOVA revealed a significant effect of Condition, *F*(2,81) = 7.22, *P*<.01, *η_p_^2^* = .15 and a significant main effect of Age Group, *F*(1,81) = 5.16, *P*<.03, *η_p_^2^* = .06, which were qualified by the predicted condition by age group interaction, *F*(2,81) = 3.79, *P*<.03, *η_p_^2^* = .09. The interaction remained significant when we included as covariates depressed mood (on the BDI) and trait anxiety (on the STAI) - variables known to be associated with AMT specificity, *F*(2, 79) = 3.55, *P*<.04, *η_p_^2^* = .08.

To deconstruct this cross-over age group by condition interaction we examined the effect of condition for each age group separately. For the younger participants, one-way ANOVA revealed the hypothesized significant effect of condition, *F*(2,42) = 16.17, *P*<.001, *η_p_^2^* = .43. Post hoc Scheffe tests indicated that the Color Stroop group retrieved significantly fewer specific memories than both the Control Stroop, *P*<.01, Cohen's *d* = .84, and No Stroop, *P*<.001, *d* = 1.14, groups, who did not differ significantly from each other, *P*>.10, thus providing the anticipated support for an effect of ego-depletion in the younger sample. In contrast, for the older group there was no significant effect of condition, F<1, all post hoc paired Scheffe tests, Ps>.50, thus providing no support for ego-depletion effects in the older participants. None of these effects was altered by the inclusion of depressed mood or trait anxiety as covariates.

## Discussion

We hypothesized that the widely-reported effects of ego-depletion on subsequent behavioural task performance [6] would be restricted to younger participants below the age of 25. This was based on our assumption that such depletion effects arise as a function of the incomplete development of the prefrontal cortex generally assumed to characterize this age group.

To test this hypothesis, participants first completed either a self-regulation depleting Color Stroop, a non-depleting Control Stroop or No Stroop task, and thereafter their ability to recall specific memories (a task requiring self-regulation) was tested using the AMT. In line with our hypothesis, younger participants who carried out the Color Stroop task performed worse on the AMT relative to those younger participants who were assigned to the Control Stroop and No Stroop conditions, indicative of significant ego-depletion in the younger sample. For the older participants in contrast there was no support for any depletion effects. Although younger participants in the non-depletion control conditions performed better on the AMT than older participants, in line with the previous literature [12], this relationship was reversed in the depletion condition giving a significant cross-over interaction (Fig. 1).

These results tie in with the timescale of frontal lobe maturation [4] so it is plausible that ego-depletion is determined by the progression of development within this area. The prefrontal cortex (PFC) is implicated in regulatory behaviours such as cognitive control and inhibition [e.g. 18], and disruptions to this area from injury or transcranial magnetic stimulation result in difficulties exerting self control [19], [20]. Where previous work has found that inhibition and self-control improve throughout childhood and adolescence, our study further, albeit tentatively, suggests that regulatory capacity fluctuates less as people get older. It is important to note other factors that may have contributed towards the effect, such as the difference between the older and younger samples in reported difficulty and fatigue arising from the Stoop task. However, these differences are entirely in line with the hypothesis that older populations do not experience the same depletion effects as younger people. If the Stroop task differentially depleted the younger participants' self-regulatory resources relative to the older participants, then the perception of this task would also be expected to vary between the two age ranges. However, future research may benefit from exploring the effects of subjective task experience on depletion.

The crucial implication of this study is the suggestion that much of the previous work on ego-depletion [6] has been confounded by the age of the majority of samples tested, because of the marked tendency to run such experiments on college students. Indeed, the high proportion of psychology studies carried out in Western countries that rely on data from college students has led to bias and lack of generalizability of psychological theories [7].

To our knowledge, this is the first study to examine age effects with respect to self-regulation depletion. The results suggest that this widely-reported phenomenon may be restricted to younger participants. This has important implications for theories that promote susceptibility to ego-depletion as a more general feature of human cognition [e.g. 21] and suggests that far more caution is warranted until the developmental boundary conditions of this phenomenon are further elucidated.
