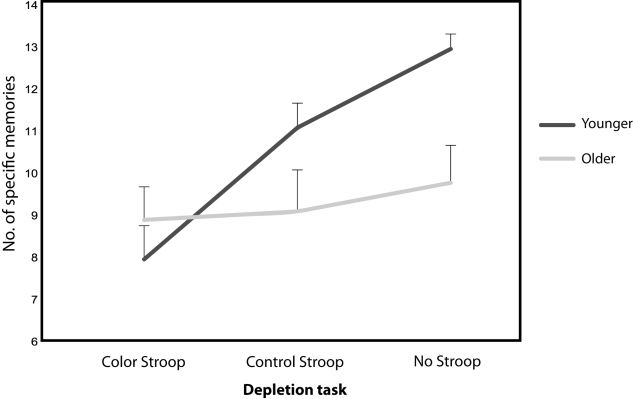# Correction: Age Shall Not Weary Us: Deleterious Effects of Self-Regulation Depletion Are Specific to Younger Adults

**DOI:** 10.1371/annotation/7e8b5c93-06a3-46c9-999b-93401d9e9cfd

**Published:** 2012-01-11

**Authors:** Theresa Dahm, Hamid Taher Neshat-Doost, Ann-Marie Golden, Elizabeth Horn, Martin Hagger, Tim Dalgleish

The correct Figure 1 can be viewed here: 

**Figure:**